# Thermodynamic Insights into the Impact of Increasing Connectivity for 2D-Lattices Based on Ising Chains

**DOI:** 10.3390/e28080894

**Published:** 2026-08-09

**Authors:** Daniel Markthaler, Kai Peter Birke

**Affiliations:** 1 Institute for Energy Efficiency in Production, University of Stuttgart, Nobelstraße 12, 70569 Stuttgart, Germany; daniel.markthaler@eep.uni-stuttgart.de; 2 Electrical Energy Storage Systems, Institute for Photovoltaics, University of Stuttgart, Pfaffenwaldring 47, 70569 Stuttgart, Germany; 3 Fraunhofer Institute for Manufacturing Engineering and Automation IPA, Nobelstraße 12, 70569 Stuttgart, Germany

**Keywords:** finite 2D-Ising model, phase transition, free energy

## Abstract

The Ising model provides a fundamental setting for investigating the emergence of phase transitions from simple interacting degrees of freedom. The current characterization study serves to investigate central requirements for phase transitions in terms of connectivity, i.e., the degree of coupled interactions between interaction sites. The impact of increasing connectivity between 1D-Ising chains mapped onto 2D-lattices with free boundary conditions were studied systematically, using exact free energy calculations. Starting from a reference system of non-interacting 1D-chains, interaction bonds between chains are introduced successively until the fully connected N×N-lattice is obtained. Two distinct construction schemes are analyzed, which differ in the connectivity of the intermediate partially coupled systems. The resulting free energies of the graphs along these paths are evaluated and compared with respect to their convergence behavior as a function of system size. We find that, despite topological differences between the schemes, strikingly, they converge to the same limiting straight line for increasing *N* when analyzed in terms of residual free energy differences. These findings provide insight into the relationship between interaction structure and thermodynamic behavior and suggest that appropriately chosen construction paths may serve as a basis for efficient extrapolation strategies toward the thermodynamic limit.

## 1. Introduction

The emergence of collective behavior from simple, short-ranged interactions remains one of the central themes in statistical mechanics. Within this context, the (Lenz–)Ising model [1,2] occupies a unique position. In contrast to the 1D-Ising model, the two-dimensional version shows, despite its minimal definition, a continuous (second-order) phase transition from an ordered (ferromagnetic or antiferromagnetic) to a disordered (paramagnetic) phase at a finite critical temperature $T_{c}$ [3,4]. For ferromagnetic magnets, $T_{c}$ would correspond to the Curie point where the permanent magnetic moment of a magnetic crystal disappears. The transition between these phases reflects the build-up of correlations (i.e., the distance over which the flip of one spin impacts another spin) and remains a key benchmark for understanding critical phenomena [5,6,7]. Below $T_{c}$, spins in the ferromagnetic Ising model tend to align (also without an external magnetic field), leading to the formation of large contiguous domains with spins of identical orientation (e.g., all “up” inside domains). However, a minor fraction of locally isolated clusters coexists, comprising spins of opposite orientation compared to the majority (i.e., all spins “down” in the clusters). This leads to a non-zero value for the average (spontaneous) magnetization per spin (*M*) which can be, depending on the orientation of the major domains, either positive or negative. By increasing the temperature, the phase transition occurs not suddenly but gradually. This is measured by the increasing fraction of the isolated clusters such that the absolute value of the order parameter, given by |M|, decreases smoothly towards zero at $T_{c}$ without a discontinuous jump as it would be the case for a first-order phase transition. A plot of the order parameter as a function of temperature defines the thermodynamic coexistence boundary curve in analogy to phase diagrams (e.g., in the representation of the density or specific volume over temperature) for homogeneous fluids. Below $T_{c}$, spontaneous symmetry breaking takes place, specified by two possible values for the order parameter of ±M which are equal in absolute value but differ in sign. The *M*-value with opposite sign would correspond to the reversed symmetry or phase reversion, i.e., a hypothetical and thermodynamically equivalent phase with reversed orientation ratios between domains and clusters. While the order parameter is zero for temperatures $T\geq T_{c}$, the correlation length diverges. At the critical temperature, fluctuations are dominant at all scales with no clear dominance of one spin orientation, which implies the development of scale-invariant and self-similar patterns. At $T_{c}$, the heat capacity curve when plotted as function of temperature is not discontinuous but shows a characteristic logarithmic divergence with “tails” to both sides of $T_{c}$. At high temperatures $T>T_{c}$, thermal fluctuations dominate, leading to rapid spin flipping such that the system progressively develops towards a collection of independent spins with a random “noisy” distribution of spin values. This can be regarded as a homogeneous “disordered” phase with M=0.

In general, phase transitions are due to a collective, cooperative behavior of a macroscopically large number of degrees of freedom [8]. It should be noted that the behavior described above refers to the 2D-Ising model in the thermodynamic limit, i.e., for an infinitely large lattice. Finite lattices, in contrast, for which the heat capacity profile stays finite at all system sizes, do not feature such a transition. However, most practical approaches rely on finite lattices and subsequent extrapolation to the thermodynamic limit. A fundamental challenge in this context is therefore to understand how such macroscopic behavior emerges from these finite systems. For the infinitely large 2D-Ising model the exact free energy is known [4] and can serve as a reference for testing finite-size-based extrapolation approaches.

From a conceptual point of view, the transition from small to large systems can be interpreted as a gradual increase in structural complexity. In graph-theoretical terms, an Ising lattice can be seen as a network of spins connected by interaction bonds. Adding a bond corresponds to introducing an additional interaction term in the Hamiltonian and, consequently, modifying both the energetic landscape and the combinatorial structure of accessible states. In this sense, the seemingly simple act of “drawing a line” between two spins has a well-defined statistical-mechanical meaning. Recent work has emphasized the importance of such structural aspects and their relation to patterns and organization in statistical systems [9]. With the present characterization work, we aim to systematically investigate the influence of increasing connectivity on the thermodynamic behavior of finite lattices and thereby gain insights into the model assumptions required to describe phase transitions. We therefore ask how to construct the fully interacting system from simpler building blocks in a way that naturally captures the correct asymptotic behavior?

We address these questions by explicitly constructing the free energy of finite quadratic Ising lattices with open, i.e., free boundary conditions (FBCs) along well-defined paths in configuration space. Starting from a reference system of a collection of independent 1D-chains, interaction bonds between chains are introduced successively involving partially connected lattices until the fully connected 2D-lattice is recovered. Importantly, such a construction is by no means unique; i.e., different sequences of bond insertions correspond to different intermediate graph topologies. Such a strategy always involves the decision about how to draw the first bond, as well as applying a strategy for how to include the remaining bonds in a particular sequence.

The present work has to be viewed in the broader context of a large and diverse set of analytical and numerical methods that have been developed over the past decades to investigate finite Ising systems and their thermodynamic-limit behavior. A good overview can be found in several specialized statistical-mechanical text books such as [10]. These methods can broadly be classified into three conceptually distinct categories. (i) A first class comprises mean-field approaches, including the classical mean-field approximation, where the immediate environment of a particular spin *i* is not explicitly resolved but the influence on *i* caused by interactions with its neighboring spins is taken into account only in averaged form, analogous to an external field. This crude approximation is relaxed in the approximation by Bethe [11], where nearest neighboring spins are treated explicitly while only the spins beyond are approximated by their average; i.e., higher-order correlations are neglected. The Cluster Variation Method, originally introduced by Kikuchi [12], expresses the entropy in terms of probability distribution functions of local clusters, thereby systematically incorporating short-range correlations beyond the Bethe approximation. (ii) A second important class of methods is provided by perturbative series expansion techniques, including high- and low-temperature expansions, finite-lattice methods [13], and linked-cluster expansions [14]. Their common principle is to start from an exactly solvable reference state and to systematically generate corrections by enumerating connected graph topologies. In high-temperature expansions, thermodynamic quantities are expressed as power series in the coupling parameter starting from the totally disordered state, whereas low-temperature expansions are formulated around the ordered ground state. Finite-lattice methods and linked-cluster expansions further organize these contributions by calculating finite connected subgraphs whose embedding numbers allow the reconstruction of bulk thermodynamic quantities in the thermodynamic limit. These approaches have become highly successful for obtaining precise series coefficients and critical properties of lattice models, particularly in combination with resummation techniques [14]. Descriptions of the critical region based on classic Landau theory [15], in contrast, which provide a phenomenological expansion of the free energy in terms of an order parameter, were qualitatively, quantitatively, and fundamentally false [7]. (iii) A third group is formed by approaches based on finite-size scaling [6,16,17], renormalization-group theory [18] and conformal field theory [19]. Rather than constructing approximations of the partition function itself, these methods exploit universal scaling behavior near criticality to infer thermodynamic-limit properties from finite systems. In particular, finite-size scaling such as the Binder cumulant technique [6] have become the standard framework for estimating critical temperatures and critical exponents, while conformal field theory provides powerful relationships between finite-size corrections and universal quantities such as scaling dimensions and the conformal charge. Closely related studies have analyzed finite-size free energy corrections in rectangular geometries and their dependence on boundary conditions and aspect ratio using conformal invariance and exact finite-size scaling results [20,21].

The present work shares with these methods the philosophy of deriving information about the infinite system from finite connected structures, but differs fundamentally in both objective and implementation. Rather than expanding the partition function or free energy as a perturbative series around a limiting temperature, we construct a sequence of graphs of increasing connectivity that continuously interpolates between independent 1D-chains and the fully connected finite 2D-lattice. Every intermediate graph corresponds to a complete thermodynamic system with an exactly computable partition function and free energy. The central quantity of interest is therefore not the contribution of an individual cluster to a formal series expansion, but the evolution of the total free energy along a prescribed connectivity pathway.

In particular, we investigate two distinct construction schemes that differ in the connectivity of these intermediate states and analyze their impact on the resulting free energy and its convergence behavior. Our results show that, although both schemes converge to the same fully interacting system, they exhibit markedly different convergence properties when analyzed as a function of system size. In particular, representations based on residual free energy differences provide a sensitive measure of convergence and allow for a systematic comparison between the two approaches. This observation highlights that the choice of construction path, i.e., the order in which interactions are introduced, is not merely a technical detail but can significantly influence the efficiency of extrapolation strategies. The broader motivation of this work is to develop methods that extract thermodynamic-limit properties from a minimal set of small, highly accurate systems. In particular, we aim to identify scaling relations that enable reliable estimates of quantities such as the critical temperature without the need for extensive large-scale simulations. Such approaches may be especially valuable for higher-dimensional systems, where exact solutions are not available and computational costs increase rapidly.

## 2. Materials and Methods

In continuation of our previous work [22,23], the finite 2D-Ising model with nearest-neighbor interactions on regular square (N×N) lattices is studied. The system size parameter *N* denotes the linear dimension, i.e., the number of spins along the *x*- and *y*-direction, and the total number of spins is given by $N_{\mathrm{spins}}=N^{2}$. Within the framework of the classic (spin 1/2) Ising model, spins are allowed to have two possible discrete values of ±1 for the spin variable *s*, corresponding to the spin-up (+1) and spin-down state (−1). By imposing FBC, the Hamiltonian *H* in zero field can be split into a contribution involving horizontal ($H_{x}$) and vertical interactions ($H_{y}$):

(1a) $$\begin{array}{cc} H & =H_{x}+H_{y} \end{array}$$

(1b) $$\begin{array}{cc} \;\;\;\;\;\;\;\;\;\;\;\;\;\;\;\;H_{x} & =-\sum_{n=1}^{N-1}\sum_{m=1}^{N}J_{i,j}\;s_{i}\;s_{j}\mathrm{with}i=k\left(m,n\right),j=k\left(m,n+1\right) \end{array}$$

(1c) $$\begin{array}{cc} \;\;\;\;\;\;\;\;\;\;\;\;\;\;\;\;H_{y} & =-\sum_{n=1}^{N}\sum_{m=1}^{N-1}J_{i,j}\;s_{i}\;s_{j}\mathrm{with}i=k\left(m,n\right),j=k\left(m+1,n\right) \end{array}$$

(1d) $$\begin{array}{cc} \;\;\;k(m,n) & =m-1+N(n-1) \end{array}$$

**Figure 1:**
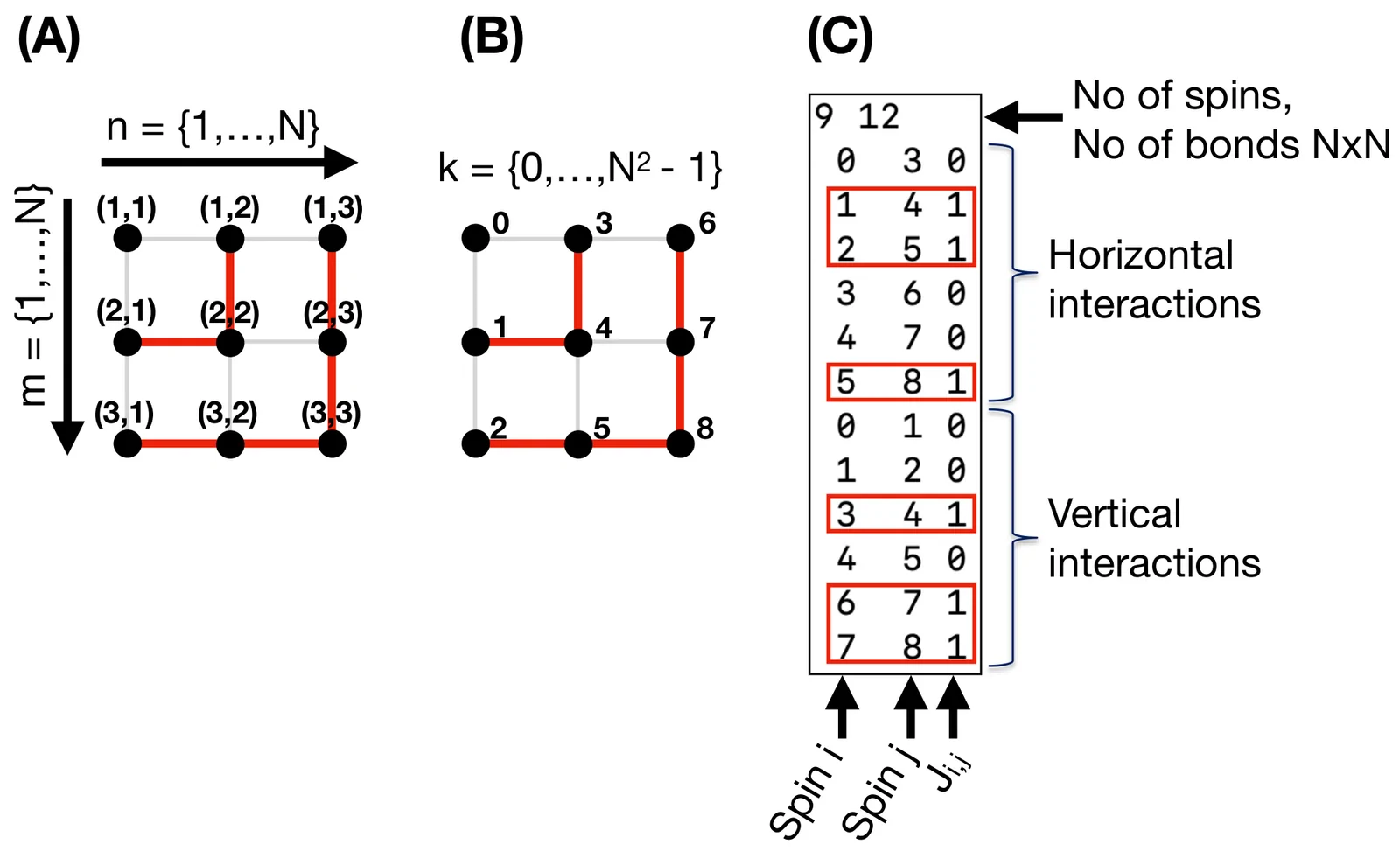
A 3×3-lattice with partially coupled interactions. Red thick lines correspond to activated interactions between neighboring spins with coupling constant $J_{i,j}=1$; grey thin lines correspond to deactivated interactions, i.e., non-coupled neighboring spins with $J_{i,j}=0$. (**A**) Double-index (*m*,*n*) scheme for spin-labeling with row index *m* and column index *n*. (**B**) Single-index scheme based on spin label *k* calculated from (*m*,*n*) positions according to Equation (1d). (**C**) Connectivity matrix used as input file for the applied free energy code (see Section 2). Numbers in the first line refer to the spin number $N_{\mathrm{spins}}=N^{2}$ and the total (i.e., maximal) number of bonds $N_{b,\max}=2N\left(N-1\right)$ in case of a fully coupled N×N-system if all nearest-neighbor interactions were activated. The following lines have three columns and denote all neighboring spin pairs *i*, *j* (both according to the single-index scheme in (**B**)) and the applied $J_{i,j}$ between them which can adopt values of 0 and 1. Activated interactions are highlighted by red boxes.

The (ferromagnetic) coupling constant $J_{i,j}$ measures the strength of interaction between neighboring spins *i* and *j* and is set to 0 or 1, in the case of non-coupled or fully coupled interactions, respectively. The function in Equation (1d) transforms the double-index (*m*,*n*) scheme to a single-index (*k*) scheme for labeling spin positions (see also Figure 1A,B). Throughout the text, the inverse temperature β is used instead of the absolute temperature *T*. For simplicity, Boltzmann’s constant $k_{B}$ is set to 1 which implies β=1/T.

Exact partition functions ($Z_{N}\left(\beta \right)$) of finite 2D-Ising lattices were computed using a polynomial-time algorithm of Karandashev et al. [24] that is based on the Fisher–Kasteleyn–Temperley algorithm and is publicly available [25]. Exploiting the fact that for planar graphs the partition function can be reduced to the Pfaffian of a suitably constructed skew-symmetric matrix, the method relies on identifying a Pfaffian orientation and evaluating the corresponding matrix determinant. This approach enables the exact calculation of the Helmholtz free energy $A_{N}\left(\beta \right)=-\ln Z_{N}\left(\beta \right)/\beta$ for arbitrary planar spin systems with pairwise interactions including lattices with partially and fully connected interactions.

Figure 1C shows the format of the connectivity matrix used as input file for the free energy code in case of a partially connected 3×3-lattice. The free energy code returns the canonical partition function $\ln Z_{N}\left(\beta \right)$ together with $\ln Z_{N}\left(\beta +\Delta \beta \right)$ and $\ln Z_{N}\left(\beta +2\Delta \beta \right)$ with $\Delta \beta =10^{-5}$. Values evaluated at β+Δβ and β+2Δβ can be used to estimate first- and second-derivative properties such as entropy and heat capacity from a finite-differences approach. The free energy code was called using the Python subprocess module. For every system size *N*, values for $A_{N}\left(\beta \right)$ at equidistantly distributed points in the interval β=[0.2,3.0] (as well as $\beta_{c}$ and $\beta_{\max}\left(N\right)$) were written to a CSV file for further analysis such that every line corresponds to a particular state along the generated path. The two characteristic values $\beta_{c}=1/T_{c}=\ln\left(1+\sqrt{2}\right)/2\approx 0.441$ [4,26] and $\beta_{\max}\left(N\right)$ denote the inverse of the critical temperature of the 2D-Ising model in the thermodynamic limit, i.e., for N→∞ and the temperature value at the individual heat capacity maximum of the finite system, respectively. The latter was estimated via the two-parameter scaling law presented in our recent work [23] (compare Equation (10a,b) therein) with $\beta_{\max}\left(N\right)=1/T_{\max}\left(N\right)$ and

(2a) $$\begin{array}{cc} T_{\max}\left(N\right) & \approx \hat{a}\;\frac{N}{N+1}+\hat{b} \end{array}$$

(2b) $$\begin{array}{c} \;\;\;\;\;\;\;\;\;\;={\hat{T}}_{c}-\frac{\hat{a}}{N+1}\mathrm{with}{\hat{T}}_{c}=\hat{a}+\hat{b} \end{array}$$

(2c) $$\begin{array}{c} \;\;\;\;\;\;\;\;\;\;\;\;\;\;\;\;={\hat{T}}_{c}\left(1-\frac{\hat{c}}{N+1}\right)\mathrm{with}\hat{c}=\hat{a}/{\hat{T}}_{c}=\hat{a}/\left(\hat{a}+\hat{b}\right) \end{array}$$

with model parameters ${\hat{T}}_{c}=2.2719\pm 0.0012$, $\hat{a}=3.0597\pm 0.0071$. This value is an extrapolated approximative value for the true $T_{c}$ that was derived using a simple and new approach based on exact heat capacity peak temperatures (from the density of states (DOS)) of a small set of very small lattices. It was obtained from optimization of Equation (2b) to exact DOS data for system sizes 2≤N≤12. Details on how this extrapolation method and the approximate value were determined are described in our recent work [23]. It should be noted that the estimated uncertainty bounds do not bracket the exact value $T_{c}=2/\ln\left(1+\sqrt{2}\right)\approx 2.2692$. The primary application of Equation (2b) in the scope of the current work, which involve calculation of the different states of a specific finite lattice at the individual peak temperature of the fully connected finite lattice, is to find reliable estimates for the heat capacity peak temperatures for finite lattices (such as N=100) for which the DOS can not be calculate directly due to computational constraints. For this application, the approximation above is sufficient. The relation according to Equation (2b) is only applicable for the *N*-dependence of the fully connected lattice, i.e., the end state of the construction path, but not for the partially connected intermediates.

**Figure 2:**
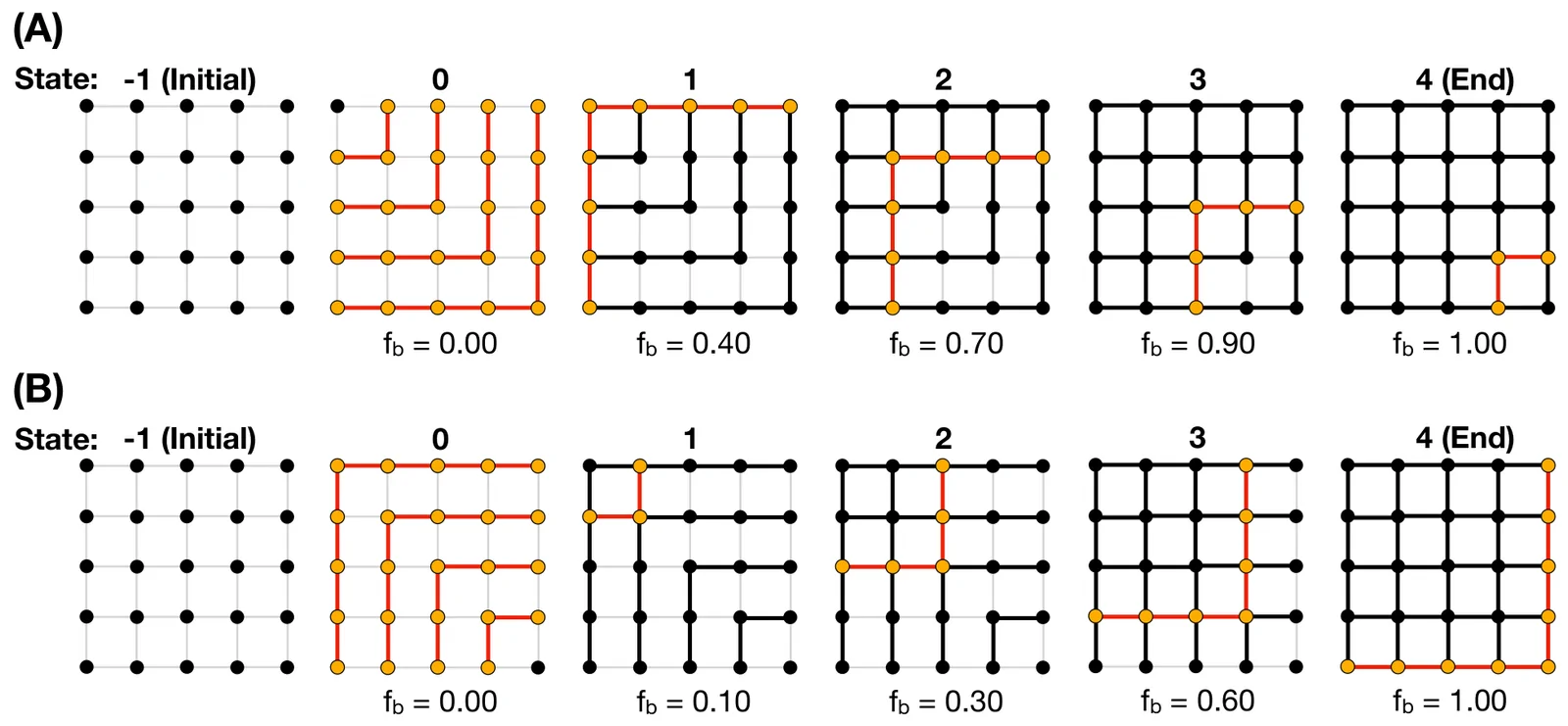
Construction schemes (**A**,**B**) as applied in this work for a lattice of size N=5 (see Section 2): independent spins for the 2D-topology of interest (=state −1), are first summarized to a set of non-interacting 1D-Ising chains (=state 0). In the following steps, successive layers of inter-chain interactions (=bonds) between neighboring chains are added sequentially, finally resulting in a fully connected graph (=state 4). Affected bonds which are added in a particular step are highlighted in red together with involved spins colored in yellow. Corresponding bond fractions $f_{b}$ according to Equation (7b) are given below each state.

In this work, two different construction schemes were studied, each corresponding to a unique free energy path. Figure 2 shows the two construction schemes (A, B) in case of a 5×5-lattice. In the initial state (−1), the $N_{\mathrm{spins}}$ non-interacting spins (with all $J_{i,j}$ = 0) of the N×N-system are summarized to a set of *N* non-interacting, i.e., independent 1D-Ising chains (=state 0) through inclusion of N(N−1) bonds. Here, the shortest chain consists of a single spin while the longest chain has a length of 2(N−1) bonds involving 2N−1 spins. The corresponding free energies for the system of uncoupled spins and chains are given by [22]:

(3a) $$\begin{array}{cc} A_{\mathrm{spins}} & =A_{N,-1}=-\frac{N^{2}}{\beta }\ln2 \end{array}$$

(3b) $$\begin{array}{cc} \;\;\;\;\;\;\;A_{\mathrm{chains}} & =A_{N,0}=A_{\mathrm{spins}}-\frac{N(N-1)}{\beta }\ln\left(\cosh\beta \right) \end{array}$$

In the following steps (=state 1 to state N−1), the remaining N(N−1) bonds between the chains are added in a sequential manner, resulting in a fully connected N×N-graph. This is in contrast to our previous approach [22] where only a single bond type was considered—i.e., all inter-chain bonds between state 1 and the end state were added simultaneously. While both tested schemes in this work are similar and identical up to state 0, they differ in the generated partially connected graphs, i.e., the intermediate states along the path to the fully connected end state.

According to Figure 2A, scheme A adds 2(N−1) bonds, i.e., (N−1) horizontal and an equal number of vertical bonds in the first step, originating from the upper left spin (1,1) towards the corresponding spins at the boundaries (numbering according to Figure 1A). Next, the procedure is repeated by inclusion of 2(N−2) bonds, originating from spin (2,2) and so forth until the remaining last two bonds are added, originating from spin (N−1,N−1). In the course of this procedure, the number of added bonds is gradually reduced from state to state, however, all initial chains of state 0 are always connected at any intermediate state.

For the alternative scheme B in contrast, the number of added bonds gradually increases from state to state: step 1: 2 added bonds; step 2: 4 added bonds; …; last step: 2(N−1) added bonds (compare Figure 2B). With each subsequent step, the next shorter, unconnected neighboring chain is simply linked at one corner spin to the chains that are already connected and it is only in the final step that all the chains are connected to one another. For both schemes, there is a total of *N* states, starting at state 0 and ending at state N−1. The relationship between both construction schemes is given by a kind of inverse symmetry: scheme B is derived from scheme A by adding the initial two inter-chain bonds to the bottom right position in the lattice (starting at spin (N−1,N−1) in state 0 in Figure 1A) and proceeding upwards to the top left (ending at spin (1,1)).

Results for $A_{N}\left(\beta \right)$ of partially and fully connected graphs calculated by the free energy code were benchmarked for small systems up to N=5 by brute force calculations where all $2^{N_{\mathrm{spins}}}$ possible configurations for each state were systematically generated, using the Python itertools module. State 0 was validated against the analytically calculated free energies for independent chains according to Equation (3b). For system sizes up to N=12, the DOS-based approach from our recent work [23] which relies on the transfer-matrix method [26,27] could be applied for validation. However, this approach is restricted to fully-connected lattices and is not applicable to graphs with partial interactions such as depicted in Figure 1.

All implementations and analyses were done using Python (version 3.10.12) in combination with standard libraries.

## 3. Results

In the following, the free energy will be typically studied on a per-spin basis ($a_{N}=A_{N}/N_{\mathrm{spins}}$) or as reduced free energy per spin ($\beta a_{N}=\beta A_{N}/N_{\mathrm{spins}}$) instead of the native free energy ($A_{N}$) as calculated by the free energy code. To assess the convergence behavior along the construction path, it is advantageous to study the residual free energy per spin. Here, the free energy contribution due to the independent spins (see Equation (3a)) is subtracted:

(4a) $$\begin{array}{cc} a_{N,i}^{\mathrm{res}}\left(\beta \right) & \equiv \frac{A_{N,i}\left(\beta \right)-A_{\mathrm{spins}}\left(\beta \right)}{N_{\mathrm{spins}}} \end{array}$$

(4b) $$\begin{array}{c} \;\;\;\;\;\;\;\;\;\;\;\;=a_{N,i}\left(\beta \right)+\frac{\ln2}{\beta }\mathrm{for}\mathrm{state}i=\left\{0,1,\ldots ,N-1\right\} \end{array}$$

where the index *i* refers to the particular state along construction path. In case of state 0, Equation (4b) in combination with Equation (3b) yields

(5) $$a_{N,0}^{\mathrm{res}}\left(\beta \right)=-\frac{1}{\beta }\left(1-\frac{1}{N}\right)\ln\left(\cosh\beta \right)$$

To make the free energy evolution of different system sizes comparable and display them in a single graph, the bond fraction is introduced. It is the ratio between the actual number of bonds ($N_{b}$) in a lattice of a given state and the maximal number of bonds ($N_{b,\max}$) for the fully connected N×N-system:

(6a) $$\begin{array}{cc} x_{b} & =\frac{N_{b}}{N_{b,\max}}\mathrm{with}N_{b,\max}=2N\left(N-1\right) \end{array}$$

(6b) $$\begin{array}{cc} \;\;\;\;\;\;\;\;f_{b} & =\frac{N_{b}-N\left(N-1\right)}{N_{b,\max}-N\left(N-1\right)}=\frac{N_{b}}{N(N-1)}-1=2x_{b}-1 \end{array}$$

Throughout this work, the rescaled bond fraction $f_{b}$ according to Equation (6b), which is 0 for state 0 and 1 for end state N−1, is used as connectivity measure for the (partially connected) graphs. For the two presented construction schemes (A, B), the values for $N_{b}$ and $f_{b}$ at state *i* can be explicitly calculated:

(7a) $$\begin{array}{cc} \;\;\;\;\;\;\;N_{b,i} & =\left\{\begin{array}{cc} N(N-1)+2iN-i(i+1) & \;\left(A\right)\\ N(N-1)+i(i+1) & \;\left(B\right) \end{array}\right. \end{array}$$

(7b) $$\begin{array}{cc} f_{b,i} & =\left\{\begin{array}{cc} \frac{2iN-i(i+1)}{N(N-1)} & \;\left(A\right)\\ \frac{i(i+1)}{N(N-1)} & \;\left(B\right) \end{array}\right.\\ \mathrm{for}\mathrm{state}i & =\{0,1,\ldots ,N-1\} \end{array}$$

### 3.1. Thermodynamic Characterization of the Construction Schemes

**Figure 3:**
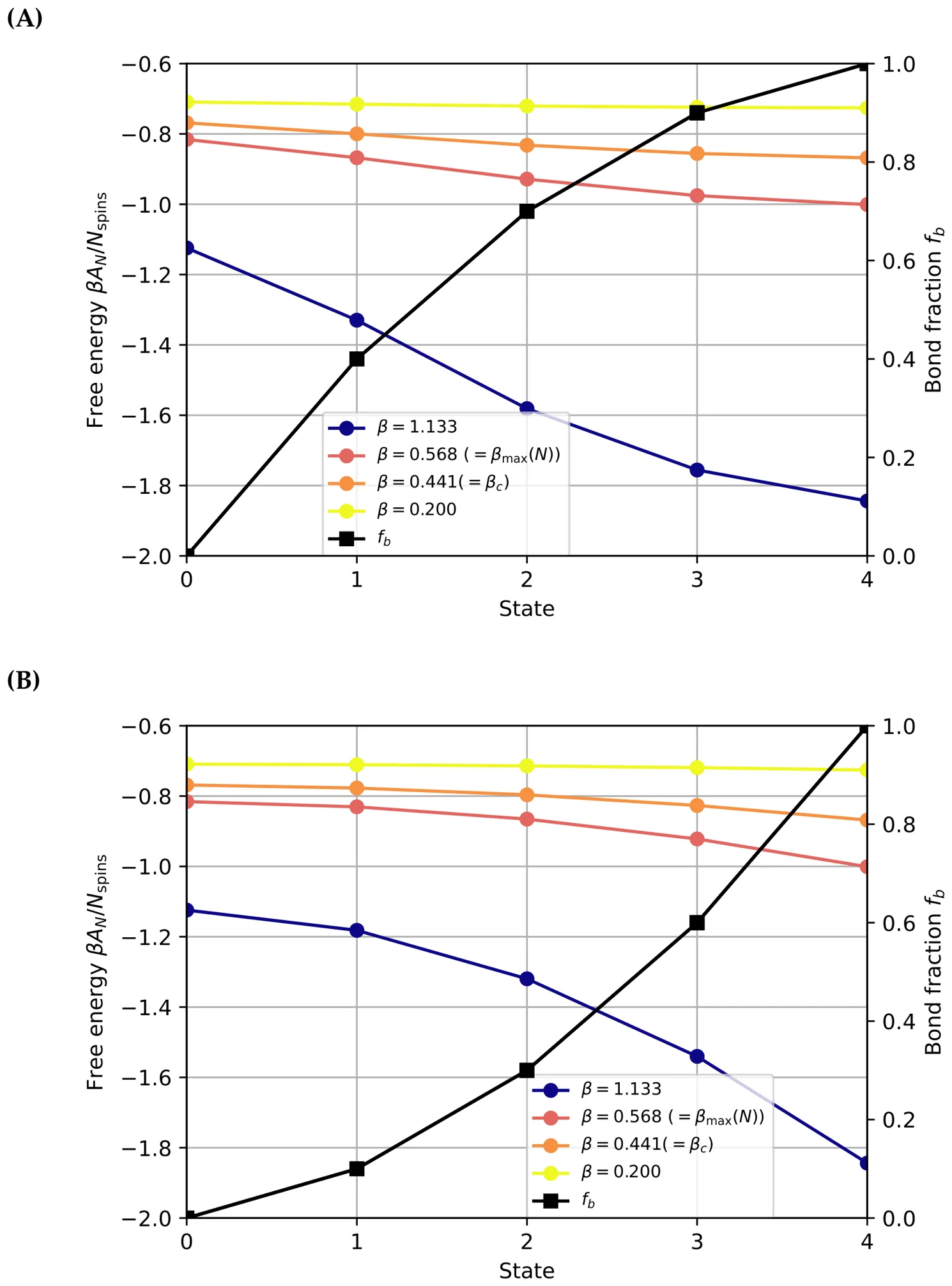
Progression of the reduced free energy per spin at five different temperatures 0.2≤β≤1.133 (left vertical axis, colored curves) and the corresponding bond fraction $f_{b}$ (right vertical axis, black curve) along the different states of the construction pathway for a lattice of size N=5 (compare Figure 2). (**A**) Construction scheme A; (**B**) construction scheme B.

Figure 3 shows the free energy evolution for the different states at various temperatures for both construction schemes in case of a 5×5-lattice. In general, the free energy declines with each further added bond, indicating increasing stability of the lattice. This variation with increasing connectivity is more pronounced at lower temperatures (higher β). While the free energies for the initial state 0 (=independent chains) and the end state N−1=4 (=fully connected 5×5-lattice) are identical at all temperatures for both schemes, it is obvious that the progression in between, i.e., the free energy path of the partially connected intermediate states, differs significantly. For scheme A, where most bonds are added at the beginning and increasingly less in the course of the construction, the corresponding variations in free energy and bond fraction (right vertical axis, black curve) are more pronounced at the start and then gradually decrease. In contrast, for scheme B, where few bonds are added at the beginning and then more bonds are added with each step, the trend is exactly the opposite, i.e., small variations in both quantities at the start, which then become increasingly pronounced towards the end.

**Figure 4:**
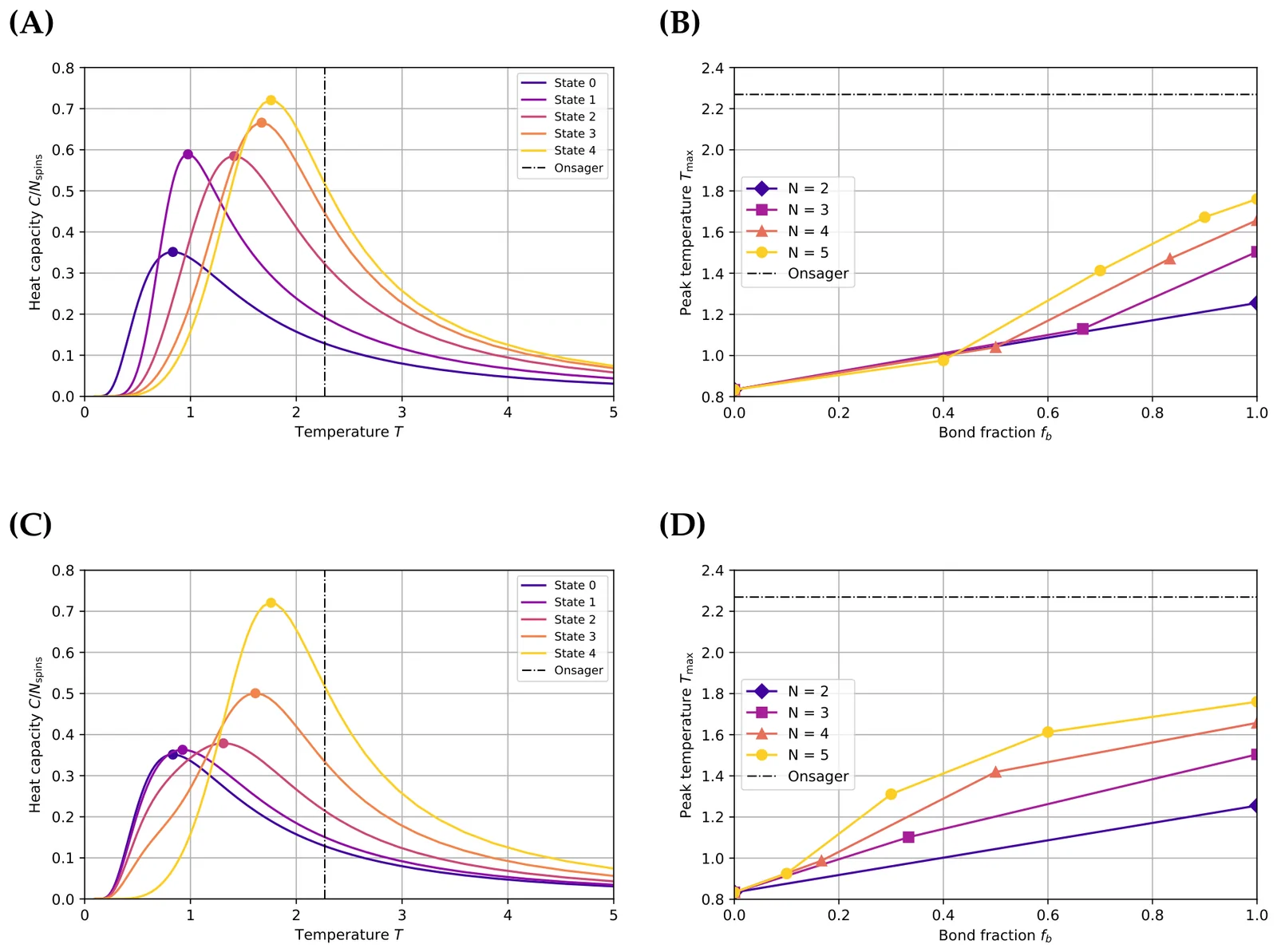
Left: Heat capacity profiles per spin $C/N_{\mathrm{spins}}$ calculated from the DOS as a function of temperature for all states along the construction pathway for a lattice of size N=5 (compare Figure 2). (**A**) Construction scheme A; (**C**) construction scheme B. Calculated heat capacity maxima are highlighted by circles. Right: Progression of the peak temperature $T_{\max}$ as a function of bond fraction for the different states along the construction pathway for lattice sizes 2≤N≤5. (**B**) Construction scheme A; (**D**) construction scheme B. $T_{\max}$ was obtained from the maximum of the corresponding heat capacity profile (compare (**A**,**C**)). The black dashed–dotted line corresponds to the critical temperature of the infinitely large lattice (“Onsager”).

Further insights into the thermodynamic behavior of the sequence of generated states can be obtained from the study of heat capacity profiles per spin ($C/N_{\mathrm{spins}}$) which are shown again for a 5×5-lattice in Figure 4A,C. Heat capacity profiles together with the peak temperature $T_{\max}$ at the heat capacity maximum were calculated from the DOS of each state (obtained from brute force calculations) as described in detail previously [23]. It can be seen that profiles for all states (including the initial state of unconnected chains) feature the characteristic unimodal shape with a single maximum. As noted earlier, in scheme A, the difference in the *C*-curves during the first step (0→1) is most pronounced compared to the subsequent steps, since most interactions are activated in this step. While for scheme B, in contrast, the difference in *C*-curves for the transition 0→1 is quite small, since the minimum number of bonds are added during this step, the differences between profiles become more pronounced with each subsequent step.

The characteristics for the schemes become further apparent by plotting the peak temperature $T_{\max}$ determined from the heat capacity profiles as a function of bond fraction for various lattice sizes as shown in Figure 4B,D. In both cases, $T_{\max}$ rises steadily as the connectivity increases, which again can be interpreted as indicator for increasing stability. For scheme A, the peak temperatures show greater variations between successive states than is the case within the sequence of states for scheme B. It is also evident that scheme B generates more points i.e., a finer resolution at small $f_{b}$ and less points towards large $f_{b}$-values compared to scheme A.

### 3.2. Convergence of the Construction Schemes

**Figure 5:**
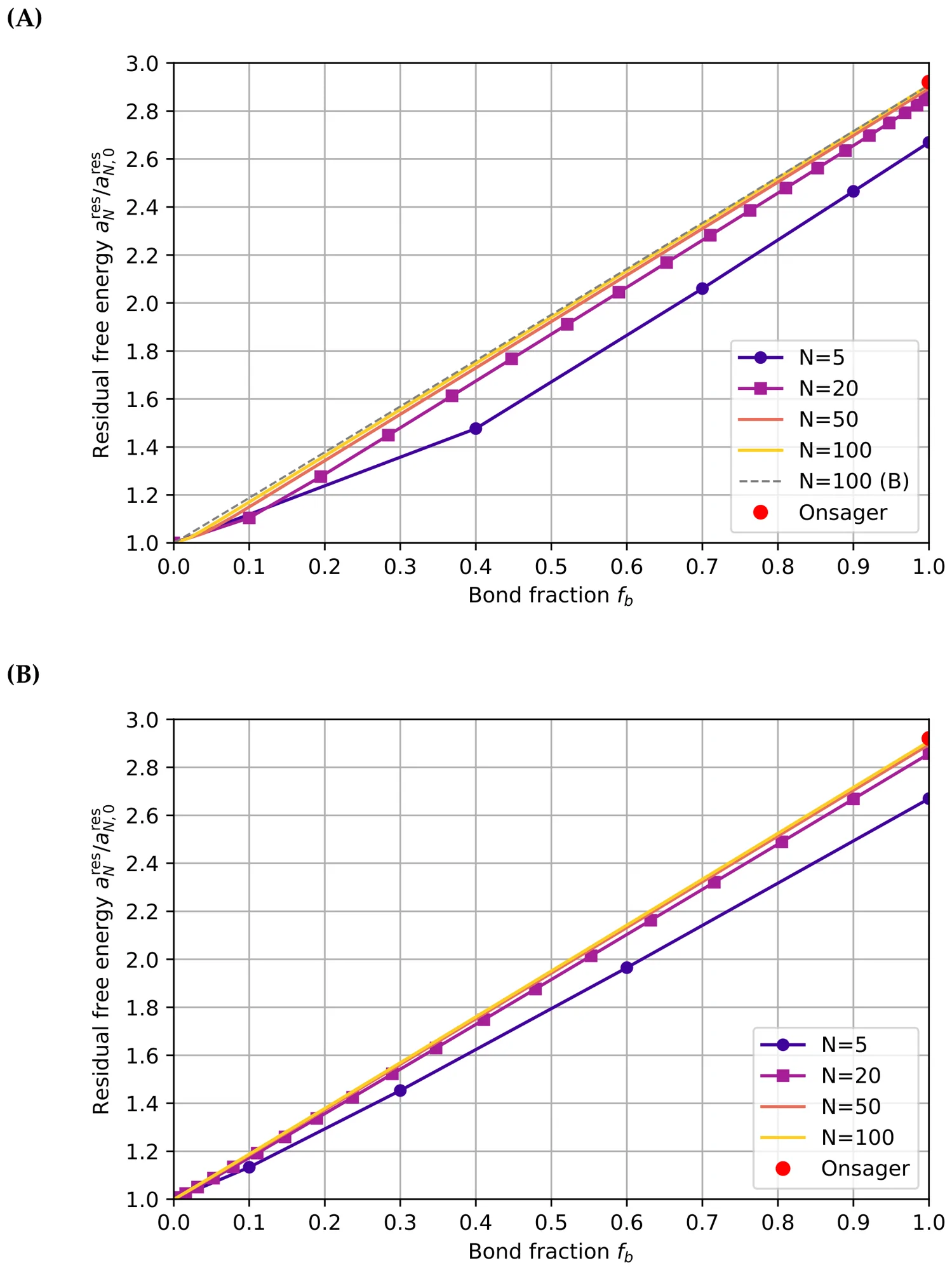
Normalized residual free energy according to Equations (4a) and (5) as function of the bond fraction $f_{b}$ for four different lattice sizes 5≤N≤100 (colored curves) evaluated at β=1.133. For the sake of clarity, markers were omitted for N≥50. (**A**) Construction scheme A; (**B**) construction scheme B. The grey dashed curve in (**A**) refers to the residual free energy for N=100 from scheme B which is shown for direct comparison. The red dot corresponds to the residual free energy of the fully connected infinitely large lattice (“Onsager”) calculated from Equation (9).

Figure 5 shows the residual free energy in normalized form as function of bond fraction for various lattice sizes at constant temperature (β=1.133). For both schemes the dependence of the isothermes on the bond fraction becomes linear as *N* increases. It can be described well by the simple approach:

(8) $${\left.\frac{a_{N}^{\mathrm{res}}\left(\beta ,f_{b}\right)}{a_{N,0}^{\mathrm{res}}\left(\beta \right)}\right|}_{N\to \infty }\approx h\left(f_{b}\right)=\hat{a}\cdot f_{b}+\hat{b}$$

with slope $\hat{a}$, offset $\hat{b}$ and the normalization constant $a_{N,0}^{\mathrm{res}}=a_{N}^{\mathrm{res}}\left(f_{b}=0\right)$ according to Equation (5). As can be seen, the linear approximation works well for both schemes even at moderate system sizes which allows robust calculation of the model parameters from only two free energy data points. This is also evident quantitatively from Table 1 if one considers the similar order of magnitude of the different error measures between both schemes at given *N*, which is overall very small. Due to convergence for increasing *N*, both schemes approach the same limiting straight line. For reference, Figure 5 also contains the residual free energy per spin in the case of an infinitely large lattice (red dot), which can be calculated from the solution according to Onsager [4]:

(9) $$\begin{array}{cc} a_{\infty } & =-\frac{\ln2}{\beta }-\frac{\ln\left(\cosh2\beta \right)}{\beta }-\frac{1}{2\pi \beta }\int_{0}^{\pi }\ln\left\{\frac{1}{2}\left(1+\sqrt{1-k^{2}\;\sin^{2}\varphi }\right)\right\}d\varphi \\ \mathrm{with}\;\;k & =\frac{2\sinh2\beta }{\cosh^{2}2\beta } \end{array}$$

Despite the good linear approximation even for finite *N*, it can be seen that the largest system size considered (N=100) still shows a deviation towards the limiting value for N→∞ at the considered temperature. Obviously, this deviation is identical for both schemes, since the endpoints of the fully connected system at $f_{b}=1$ are identical.

**Table 1:** Linear approximation of the normalized residual free energy curves according to Equation (8) for the free energy data presented in Figure 5 and Figure 6, evaluated for lattice sizes 5≤N≤100. Data in the upper row refer to β=1.133; data in the lower row refer to heat capacity peak temperatures $\beta_{\max}\left(N\right)$ according to Equation (2b). Model parameters $\hat{a},\hat{b}$ were calculated from two points at $f_{b}=0.5$ (where for each *N*, the point of both schemes which is closest to $f_{b}=0.5$ was selected) and $f_{b}=1.0$. Columns contain data for both construction schemes A and B in the form A/B for direct comparison. Metrics for assessing the quality of the linear approximation, coefficient of determination ($R^{2}$) and root mean square error (RMSE), were evaluated for the full range of bond fractions $f_{b}=\left[0.0,\;1.0\right]$.

N	β	$\hat{a}\left(A/B\right)$	$\hat{b}\left(A/B\right)$	$R^{2}\left(A/B\right)$	RMSE (A/B)
5	1.133	1.9881/1.7607	0.6814/0.9088	0.9471/0.9941	$1.426\;\times \;10^{-1}$/$4.677\;\times \;10^{-2}$
20	1.133	1.9745/1.8818	0.8823/0.9751	0.9980/0.9997	$2.710\;\times \;10^{-2}$/$1.063\;\times \;10^{-2}$
50	1.133	1.9444/1.9053	0.9596/0.9897	0.9998/0.9999	$7.516\;\times \;10^{-3}$/$4.255\;\times \;10^{-3}$
100	1.133	1.9331/1.9130	0.9747/0.9947	1.0000/1.0000	$2.919\;\times \;10^{-3}$/$2.154\;\times \;10^{-3}$
5	0.5675	1.8136/1.6139	0.6998/0.8995	0.9401/0.9906	$1.364\;\times \;10^{-1}$/$5.359\;\times \;10^{-2}$
20	0.4703	1.6568/1.5704	0.8842/0.9706	0.9965/0.9992	$2.943\;\times \;10^{-2}$/$1.361\;\times \;10^{-2}$
50	0.4521	1.5720/1.5392	0.9554/0.9882	0.9997/0.9999	$7.784\;\times \;10^{-3}$/$5.230\;\times \;10^{-3}$
100	0.4461	1.5421/1.5261	0.9781/0.9941	1.0000/1.0000	$2.887\;\times \;10^{-3}$/$2.524\;\times \;10^{-3}$

**Figure 6:**
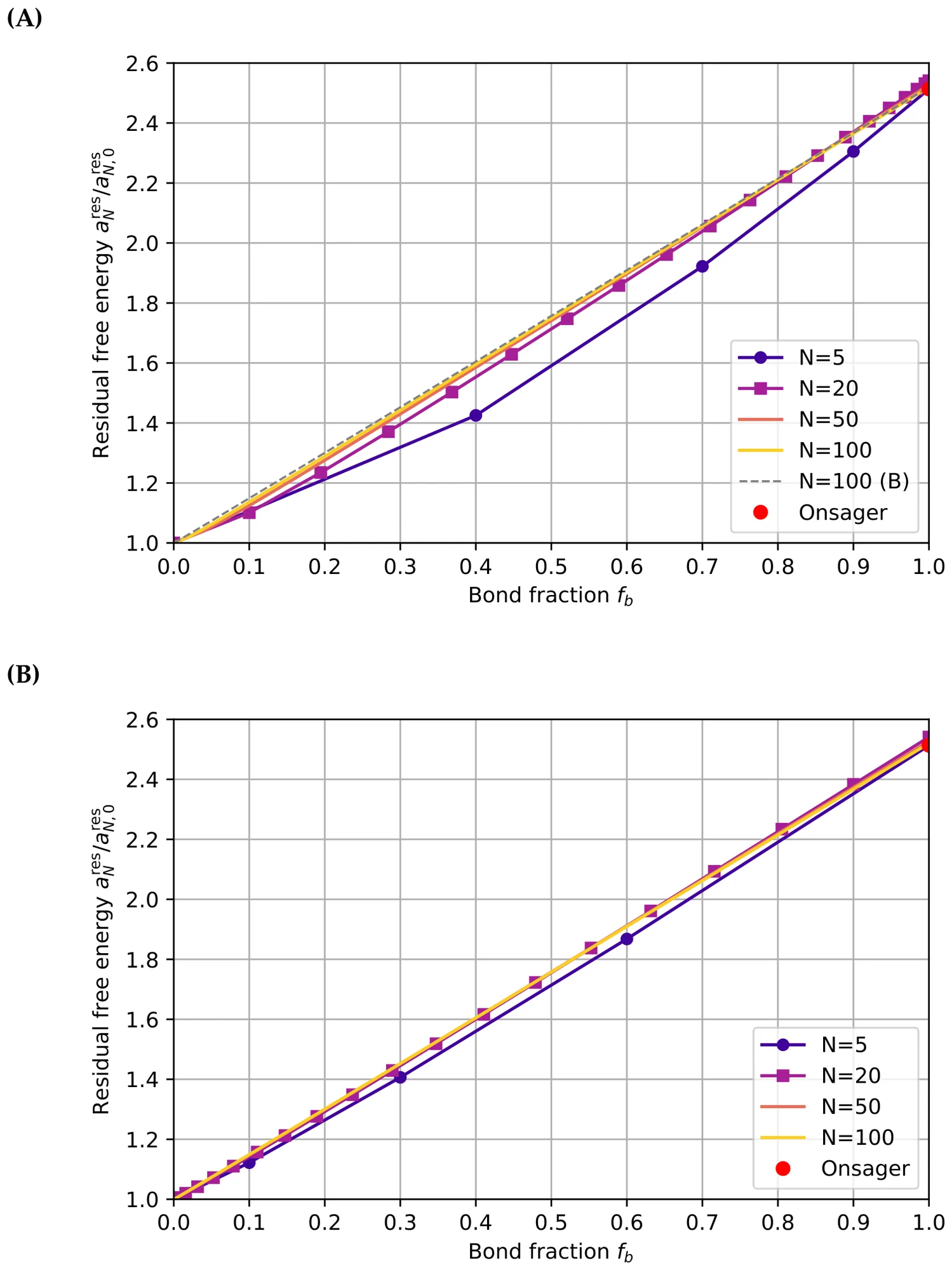
Normalized residual free energy according to Equations (4a) and (5) as function of the bond fraction $f_{b}$ for four different lattice sizes 5≤N≤100 (colored curves). For the sake of clarity, markers were omitted for N≥50. (**A**) Construction scheme A; (**B**) construction scheme B. Residual free energy values were evaluated at the individual heat capacity peak temperature $\beta_{\max}\left(N\right)$ of the underlying finite lattice according to Equation (2b). The grey dashed curve in (**A**) refers to the residual free energy for N=100 from scheme B which is shown for direct comparison. The red dot corresponds to the residual free energy of the fully connected infinitely large lattice (“Onsager”) calculated from Equation (9) at $\beta_{c}=\ln\left(1+\sqrt{2}\right)/2\approx 0.441$.

The observed behavior is also valid for other temperatures, as can be seen in Figure 6. In contrast to the previous case, the various residual free energy curves were not evaluated at a single β but at (slightly) different values, given by the individual heat capacity peak temperatures $\beta_{\max}\left(N\right)$ for each lattice size *N* according to Equation (2b). This means, as the lattice size increases, the temperature approaches the critical temperature $\beta_{c}\approx 0.441$ of the infinitely large lattice. Again, curves for both schemes can be well approximated by a straight line, where convergence in case of scheme B occurs even faster (i.e., at lower *N*) compared to the previous lower temperature. In addition, the deviation from the theoretical point of the infinite lattice is significantly smaller at this temperature. Comparison of the parameters of the linear model in Table 1 at different temperatures demonstrate that the offset $\hat{b}$ approaches unity with increasing *N* for both schemes and independent of temperature, while the slope $\hat{a}$ is temperature-dependent.

An even more sensitive quantity than the residual free energy $a_{N}^{\mathrm{res}}\left(f_{b}\right)$ to assess differences in convergence behavior between the two tested construction schemes is its slope, i.e., the difference in residual free energy between consecutive states relative to the corresponding change in bond fraction:

(10) $$\frac{\Delta a^{\mathrm{res}}}{\Delta f_{b}}=\frac{a_{N,i+1}^{\mathrm{res}}\left(\beta \right)-a_{N,i}^{\mathrm{res}}\left(\beta \right)}{f_{b,i+1}-f_{b,i}}$$

**Figure A1:**
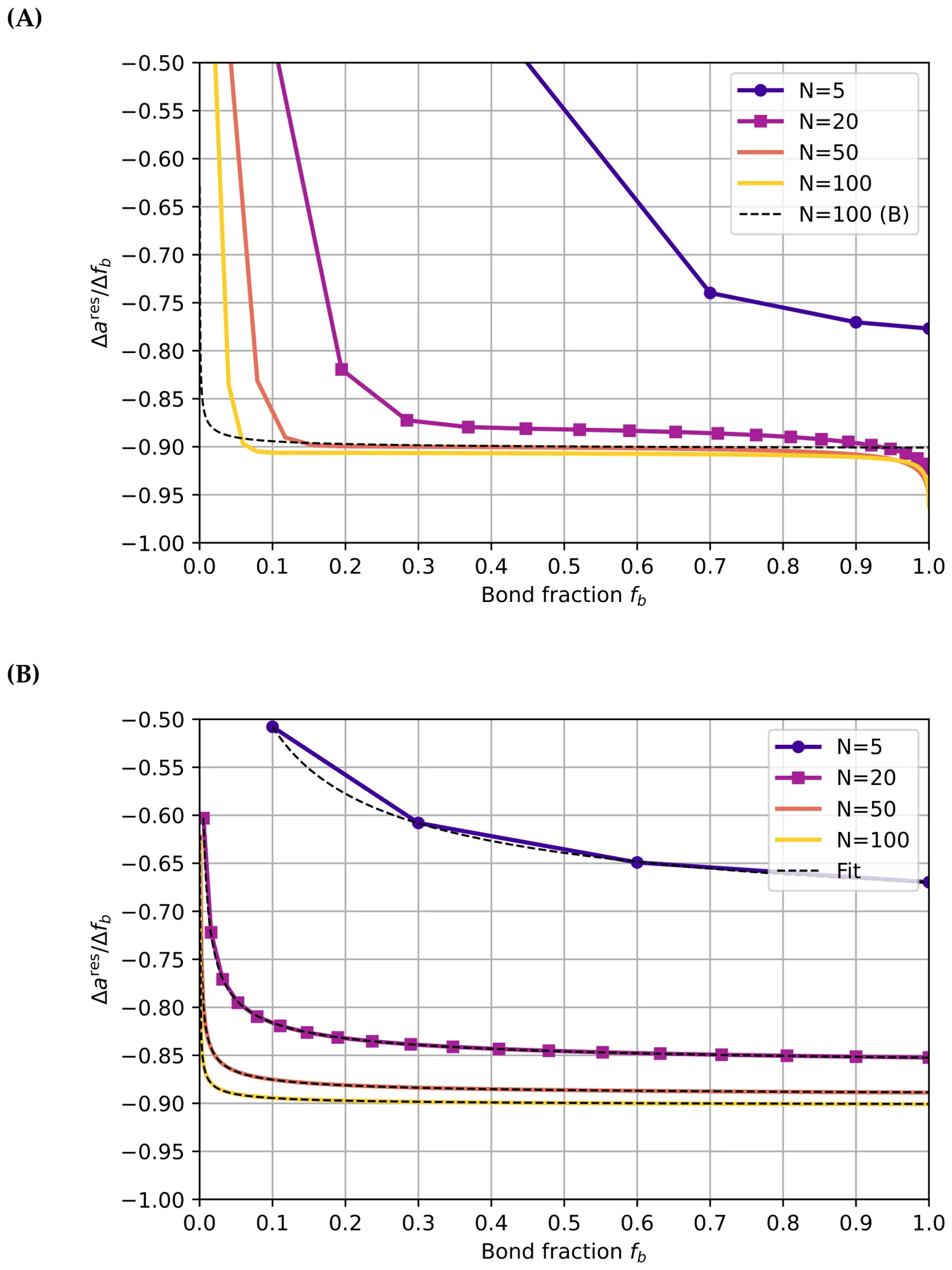
Convergence behavior of the differential residual free energy difference $\Delta a^{\mathrm{res}}/\Delta f_{b}$ according to Equation (10) as function of the bond fraction $f_{b}$ for four different lattice sizes 5≤N≤100 (colored curves) evaluated at β=1.133. For the sake of clarity, markers were omitted for N≥50. (**A**) Construction scheme A; (**B**) construction scheme B. The black dashed curve in (**A**) refers to the differential residual free energy difference for N=100 from scheme B which is shown for direct comparison. The black dashed curves in (**B**) refer to fits according to Equation (A2). Corresponding optimized model parameters are given in Table A2.

**Figure A2:**
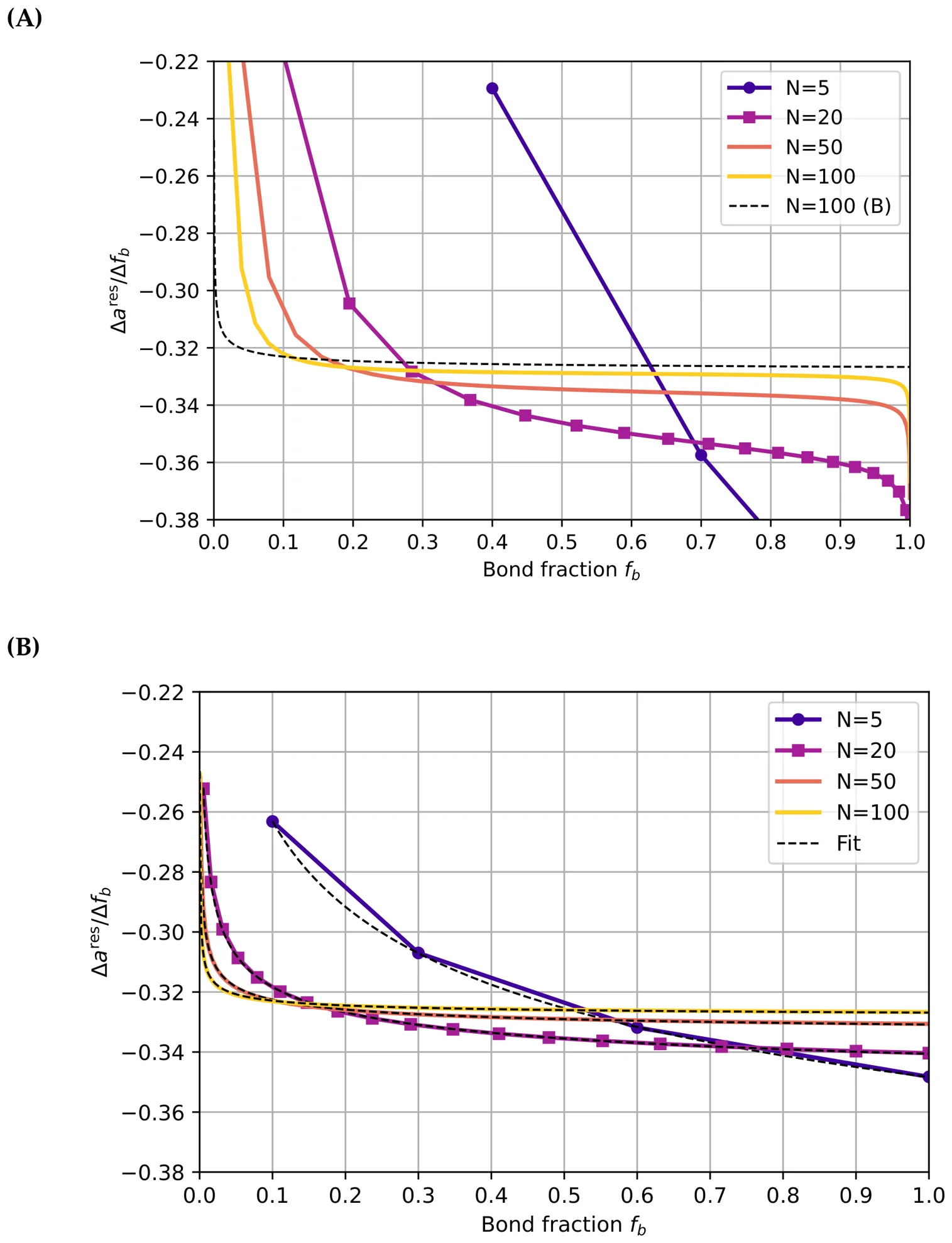
Convergence behavior of the differential residual free energy difference $\Delta a^{\mathrm{res}}/\Delta f_{b}$ according to Equation (10) as function of the bond fraction $f_{b}$ for four different lattice sizes 5≤N≤100 (colored curves). For the sake of clarity, markers were omitted for N≥50. (**A**) Construction scheme A; (**B**) construction scheme B. Residual free energy values were evaluated at the individual heat capacity peak temperature $\beta_{\max}\left(N\right)$ (according to Equation (2b)) of the underlying finite lattice. The black dashed curve in (**A**) refers to the differential residual free energy difference for N=100 from scheme B which is shown for direct comparison. The black dashed curves in (**B**) refer to fits according to Equation (A2). Corresponding optimized model parameters given in Table A2.

The difference in convergence behavior is shown in Appendix A.1. In contrast to scheme A, scheme B the differential $\Delta a^{\mathrm{res}}/\Delta f_{b}$ can be accurately approximated using a generalized negative power function with three parameters (compare Equation (A2)). From Figure A1 and Figure A2 it is evident that after an initial transient, the slope quickly approaches a constant value which emphasizes the linear approximation for $a_{N}^{\mathrm{res}}\left(f_{b}\right)$ used above. This inflection point shifts towards smaller $f_{b}$-values as *N* increases. For scheme B, a constant slope can definitely be assumed for $f_{b}\geq 0.5$ even for small *N*. This choice was taken into account when calculating the linear model parameters in Table 1. For scheme A, the situation is somewhat more complex, as the slope decreases again for high bond fractions (see Figure A1A and Figure A2A). However, as *N* increases, this decrease shifts towards increasingly higher $f_{b}$-values, meaning that the assumption of a constant slope is increasingly well-fulfilled here as well.

## 4. Discussion

**Figure A3:**
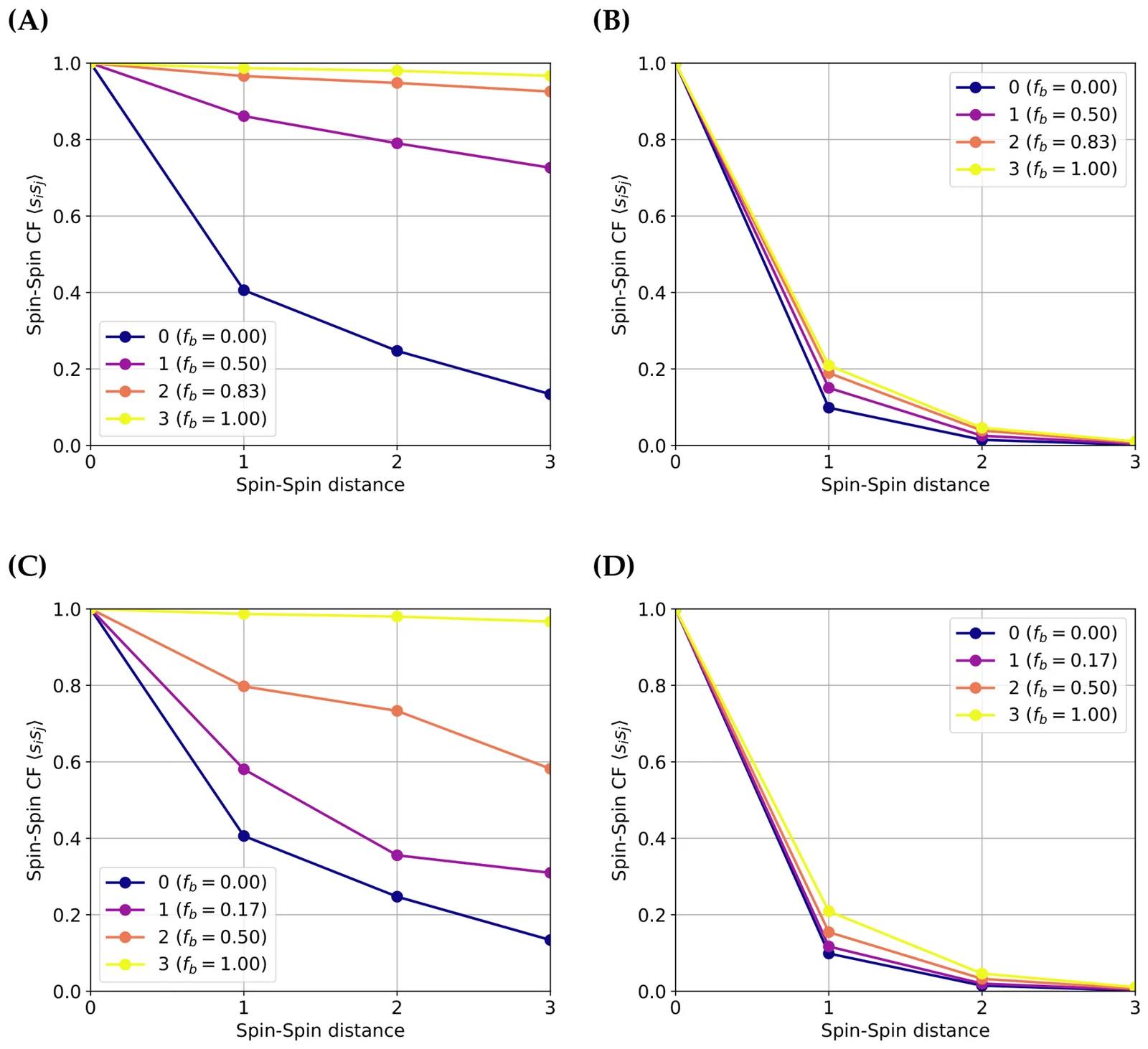
Row spin–spin correlation functions (CF) as a function of spin–spin distance for all states along the construction pathway for a lattice of size N=4 at low temperature (β=1.133, left side) and high temperature (β=0.2, right side). Upper row: (**A**,**B**) Construction scheme A; lower row: (**C**,**D**) construction scheme B.

One of the central properties of the classical 2D-Ising model is that, despite its simplicity, it exhibits a phase transition in the thermodynamic limit. A particular model requirement for this phase transition is the interaction between all directly neighboring interaction sites, i.e., spins. In this work, we aimed to understand and characterize how an increasing connectivity as measured by the bond fraction between initially non-interacting 1D-Ising chains affects the thermodynamic behavior. For this purpose, two sequential construction schemes (A, B) were investigated that gradually transform the system of independent chains into a fully interacting finite 2D-lattice through increasing connectivity, i.e., inter-chain interactions. Although the two schemes are structurally similar, they show differences in convergence behavior in terms of (differential) residual free energies as function of bond fraction. The different convergence behavior of the two construction schemes appears to be closely related to the development of spin–spin correlations in the intermediate partially connected lattices. As illustrated in Figure A3 for a lattice of N=4 system (calculated from brute force calculations), both schemes exhibit increasingly long-ranged correlations as the connectivity is progressively increased, reflecting the gradual emergence of cooperative behavior. At high temperatures, the correlations decay rapidly for both schemes, as expected. At lower temperatures, however, the intermediate states generated by scheme A exhibit noticeably longer-ranged correlations than the corresponding states of scheme B at comparable bond fractions. Consequently, newly introduced interaction bonds in scheme A interact with an already more strongly correlated environment, leading to larger cooperative contributions to the free energy increments. In contrast, the more localized correlations observed for scheme B result in smoother and more regular free energy changes.

An interesting observation is that, despite the markedly different connectivities of the intermediate graphs generated by schemes A and B, the (normalized) residual free energy depends (approximately) linearly on the bond fraction and both schemes approach the same linear relationship with increasing lattice size. While the local topological differences between the schemes remain relevant for finite systems, the influence of the specific construction pathway progressively diminishes towards the thermodynamic limit. This observation suggests that the asymptotic free energy evolution is governed primarily by the overall degree of connectivity rather than by the particular sequence in which interactions are introduced. A possible interpretation is that the free energy contribution associated with each newly activated interaction becomes increasingly determined by the average environment of the lattice rather than by local details of the partially connected graph.

The observed behavior shows some conceptual resemblance to the process of bond percolation, where the progressive occupation of interaction bonds changes the connectivity of the underlying graph and eventually leads to the emergence of system-spanning clusters above a critical occupation probability $p_{c}$ [10,28]. In diluted ferromagnets, this percolation threshold probability represents the minimum concentration of interaction bonds required to set in long-range cooperative order. However, the present work differs fundamentally from classical percolation theory. In bond percolation, bonds are occupied randomly and independently according to a probability *p*, and thermodynamic quantities are obtained after configurational averaging over quenched disorder. In contrast, the interaction networks considered here are generated by deterministic construction schemes, in which every intermediate graph is uniquely defined and its partition function and free energy are calculated exactly. Consequently, the objective is not to determine a percolation threshold or cluster-size distribution, but to investigate how the sequential establishment of connectivity influences the thermodynamic evolution of finite Ising systems.

The presented construction schemes may also formally resemble disordered Ising models such as spin-glasses [29,30], since the coupling constants $J_{i,j}$ assume values of either zero or $J_{i,j}=J=1$, depending on whether a particular interaction bond has already been introduced. However, in contrast to spin-glass models, the couplings are neither random nor frustrated but are prescribed deterministically according to the selected construction pathway. The resulting partially connected lattices therefore represent intermediate graph topologies rather than realizations of quenched disorder. Nevertheless, the close relationship to diluted and disordered Ising models suggests that extending the present framework to random or thermally annealed interaction networks could provide an interesting direction for future work.

Whether the observed linear behavior of the residual free energies as function of $f_{b}$ also applies in 3D remains to be tested. Although the present study is restricted to finite 2D-Ising lattices, the underlying construction concept is, in principle, not limited to two dimensions. A possible extension to 3D-systems would again start from independent 1D-chains, which are subsequently coupled to form independent 2D-layers before finally introducing interactions between neighboring layers until the fully connected cubic lattice is obtained. Such a hierarchical construction may provide a systematic framework for studying the emergence of cooperative thermodynamic behavior, however, several important challenges are expected. In contrast to the 2D-case, where exact thermodynamic reference data are available for small systems and the thermodynamic limit, no comparable analytical solutions exist in 3D. Consequently, accurate estimates of the DOS would have to be obtained numerically, for example using flat-histogram Monte Carlo techniques such as Wang–Landau sampling [31,32]. Furthermore, the number of possible intermediate graph topologies and construction paths increases substantially. Future work will therefore focus on identifying suitable construction schemes that preserve the favorable convergence behavior observed in the present study while remaining computationally tractable for 3D-lattices.

The temperature-dependence of the (converged) parameters as part of the models used for approximating $a_{N}^{\mathrm{res}}$ and $\Delta a^{\mathrm{res}}/\Delta f_{b}$ as function of $f_{b}$ (see Table 1 and Table A2), suggests that the construction schemes might be applied to extrapolate the critical temperature. More generally, the approach could be of interest for studying cooperative effects in partially connected or evolving lattice systems, such as adsorption processes where the degree of connectivity strongly influences the macroscopic thermodynamic response.

While the work is primarily intended as a characterization study, another motivation in line with our previous work [22], is the development of a simple analytical framework for approximating the free energy of (finite) lattices in 2D and 3D. In this regard, it is important to note that although the partition function (or equivalently free energy) of the finite FBC-lattice can be represented as a (large) matrix, no convenient closed-form expression exists [33,34]. Using scheme B as a basis, the following design approach could be proposed for the free energy of the finite square lattice:

(11) $$\begin{array}{cc} A_{N} & \approx A_{\mathrm{chains}}+\sum_{i=1}^{N-1}\Delta N_{b,i}\;\omega_{i}\;\Delta a_{b,i}\\ \; & =A_{\mathrm{chains}}+2\omega_{1}\;\Delta a_{b,1}+\ldots +2\left(N-1\right)\omega_{N-1}\;\Delta a_{b,N-1}\\ \mathrm{with}\;\;\; & \sum_{i=1}^{N-1}\Delta N_{b,i}=N\left(N-1\right),\sum_{i=1}^{N-1}\omega_{i}=1 \end{array}$$

where $\Delta N_{b,i}=2i$ denotes the number of added inter-chain bonds for a particular step *i* and $A_{\mathrm{chains}}$ is taken from Equation (3b). The weighting factors $\omega_{i}$ represent potential degrees of freedom for the approach. In our previous work [22], a simpler route was followed by considering only a single bond type which is equivalent to adding all inter-chain bonds simultaneously. The major challenge lies in accurately estimating (or modeling) the free energy per bond $\Delta a_{b,i}=\Delta A_{N}\left(i-1\to i\right)/\Delta N_{b}\left(i-1\to i\right)$. The calculation for the very first step is straightforward:

(12a) $$\begin{array}{cc} \;\;\Delta a_{b,1} & =\frac{\Delta A_{N}\left(0\to 1\right)}{2}=-\frac{1}{2\beta }\ln\left(\frac{Z_{4}^{1D,\mathrm{PBC}}}{Z_{1}^{1D,\mathrm{FBC}}\cdot Z_{3}^{1D,\mathrm{FBC}}}\right) \end{array}$$

(12b) $$\begin{array}{c} =-\frac{\ln\left(\cosh\beta \right)}{\beta }-\frac{1}{2\beta }\ln\left(1+\tanh^{4}\beta \right) \end{array}$$

This is based on the partition functions for 1D-chains with free (FBC) and periodic boundary conditions (PBC):

(13a) $$\begin{array}{cc} Z_{N}^{1D,\mathrm{FBC}} & =2^{N}{\left(\cosh\beta \right)}^{N-1} \end{array}$$

(13b) $$\begin{array}{cc} \;\;\;\;\;Z_{N}^{1D,\mathrm{PBC}} & =2^{N}\cosh^{N}\beta \;\left(1+\tanh^{N}\beta \right) \end{array}$$

The prefactor of 1/2 in Equation (12a) takes into account that two bonds are added for this transition.

**Figure 7:**
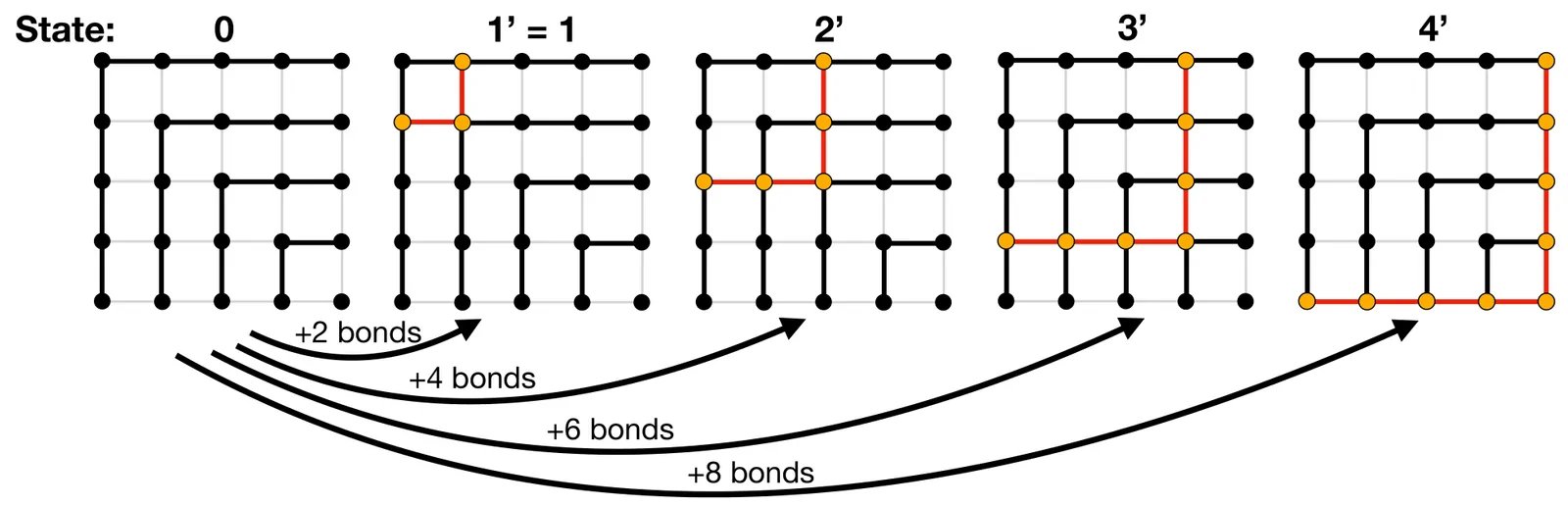
Impact of isolated inter-chain bonds added at each stage in case of scheme B for a lattice of size N=5 (compare Figure 2B). Affected bonds which are added in a particular step are highlighted in red together with involved spins colored in yellow.

Unfortunately, calculation of the remaining contributions in an analogue simple manner is (to the best of the author’s knowledge) not possible as will be discussed in more detail in the following. Figure 7 schematically illustrates, in case of scheme B, the principle for adding inter-chain bonds independently of one another, using a lattice size of N=5. This isolated addition is in contrast to the cumulative approach discussed so far, in which bonds were added one after another, including the predecessors bonds from previous steps (compare Figure 2B). To distinguish the resulting states from those of the previous schemes, they are marked with a prime (e.g., $1^{\prime }$). Quantitative comparison between the free energy contributions in the “isolated” and the “cumulative” approach are given in Table 2 for a series of lattice sizes at two temperatures. Here, the contribution is measured as free energy difference per added bond between states i,j in the construction schemes. The findings can be summarized as follows:

- The free energy per bond (at constant temperature) is not constant for all transitions for either of the two schemes (“isolated” and “cumulative”).
- The free energy contribution for a specific transition (i.e., a specific bond type) is independent of system size, for both the isolated and cumulative scheme. For example, the free energy difference associated with the transition $0\to 1^{\prime }=1$ is identical (at constant temperature) for all *N*. However, when compared with one another, these values differ between the schemes (compare e.g., $0\to 2^{\prime }$ with 1→2).
- The free energy per bond in the presence of already existing inter-chain bonds from previous steps (cumulative scheme) is always more negative (higher absolute value) compared to the corresponding value from the isolated scheme. The discrepancy between corresponding values for the two schemes increases along the construction path the more bonds are added per step.
- The *N*-dependence of the different bond types along the sequence (such as $0\to 1^{\prime },0\to 2^{\prime },\ldots$ in case of the isolated scheme or 0→1,1→2,… in case of the cumulative scheme) is significantly more pronounced for the cumulative scheme, especially at low temperature (high β). For the isolated scheme this system size dependence is weak.

**Figure 8:**
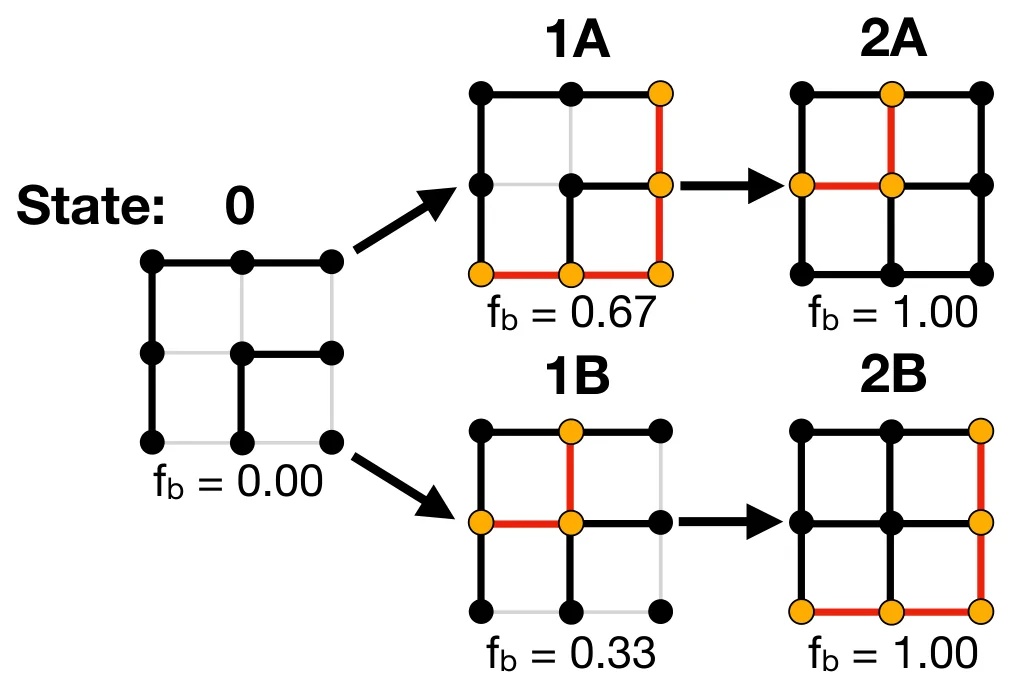
Visualization of the path-dependence of the free energy contributions associated with inter-chain bonds for a lattice of size N=3. Upper branch corresponds to construction scheme A, lower branch to construction scheme B. The end states 2A and 2B are identical. Affected bonds which are added in a particular step are highlighted in red together with involved spins colored in yellow. Corresponding bond fractions $f_{b}$ according to Equation (7b) are given below each state.

The central point is that the free energy contribution of an added bond obviously depends on the context, i.e., on the pre-existing interaction network to which the bond is added, indicating non-additive (cooperative) effects. Even though the Hamiltonian is additive and can be expressed as sum of different components (such as a horizontal ($H_{x}$) and vertical ($H_{y}$) component according to Equation (1a–c)), this does in general not apply on a free energy level which depends on the current state of connectivity of the lattices. Adding a particular inter-chain bond type in isolated form to a graph does not have the same effect on the free energy compared to the situation when the chains are already connected to some degree. The corresponding changes in the DOS will be different for the two situations; i.e., the energy levels and their populations of the accessible states will differ. As a consequence, if one would use the free energy values of the isolated scheme as the basis for $\Delta a_{b,i}$ in Equation (11), the problem would be shifted to the appropriate adjustment of the weights $\omega_{i}$, which are generally considered to be dependent on β and *N*. The origin of the difficulty is that free energy is a global system property which depends on the total volume of the available phase space [35]. This implies that it can only be exactly decomposed for independent, i.e., non-coupled, subsystems such as non-interacting spins or chains (see Equation (3a,b)). For a sequence of bond insertions (i.e., interaction changes), the free energy contribution associated with a given interaction depends on the specific construction pathway, i.e., on the order in which the interactions are introduced. This aspect is also clearly shown in Figure 8 together with Table 3 for both construction schemes (A, B) in the case of a 3×3-lattice. It can be seen that the order in which the two bond types are added is essential. It should be noted that although the free energy is a state function and as such path-independent, particular free energy components such as different bonds are not and are thus only defined for a specific path [35]. It should be clearly stated that this path-dependence is not a new finding but is a well-known property for free energy calculations. However, it is important to illustrate this property in the specific context of the proposed construction schemes and to highlight its practical implications for the development of an approximate analytical free energy expression for finite Ising lattices according to Equation (11).

The idea of constructing the free energy of a finite 2D-Ising lattice from a sequence of local interaction contributions is conceptually related to earlier approximate approaches based on hierarchical or cluster-based descriptions of interacting systems. In particular, Bethe’s approximation [11] and the later Cluster Variation Method developed by Kikuchi [12], which were originally developed for the description of alloys and are formulated in terms of approximate probability distributions or cluster entropies, have to be acknowledged. In contrast to these methods, the approach presented above, explicitly follows a sequential graph-construction perspective in which interaction bonds are introduced stepwise and the associated free energy changes are evaluated directly. This distinction is important in light of the existing literature on rectangular Ising systems with free boundaries [20,21,36]. The finite-size corrections are known to contain bulk ($A_{\mathrm{bulk}}\propto N^{2}$), surface ($A_{\mathrm{surf}}\propto N$), and corner terms ($A_{\mathrm{corner}}$), and the universal part of the critical free energy ($A_{\mathrm{uni}}$) which depends on the aspect ratio and boundary geometry: $A_{N}=A_{\mathrm{bulk}}+A_{\mathrm{surf}}+A_{\mathrm{corner}}+A_{\mathrm{uni}}$.

Our work does not attempt to re-derive these universal contributions. Instead, it asks a different question: how does the free energy change as the lattice is assembled from decoupled chains into a fully connected 2D-system? In this sense, the novelty lies in the controlled interpolation between a 1D-reference system and the final 2D-interacting lattice. The observed dependence of the free energy increments on the existing connectivity of the partially constructed graph highlights the cooperative and non-additive character of the interaction network and may provide an alternative viewpoint for future development of approximate analytical representations of finite lattice systems and extrapolation strategies.

## Figures and Tables

**Table 2:** Change in free energy per added bond ($\Delta A_{N}\left(i\to j\right)/\Delta N_{b}\left(i\to j\right)$) between states i,j for different construction steps in case of scheme B with lattice sizes 2≤N≤5. Two different temperatures β={0.2,3.0} are considered. State transitions $0\to i^{\prime }$ refer to Figure 7 where inter-chain bonds are added individually; state transitions i→i+1 between adjacent states refer to sequential construction according to Figure 2B. In each column, the free energy values for the isolated addition of a bond and the corresponding transition in the sequential scheme are listed side by side (separated by “/”) to allow for a direct comparison. The value in brackets refers to the number of added bonds per step ($\Delta N_{b}$). Since states 1 and $1^{\prime }$ are identical there is no difference between individual and sequential construction and therefore the column “$0\to 1^{\prime }=1$” contains only one entry.

N	β	$0\to 1^{\prime }=1\left(2\right)$	$0\to 2^{\prime }/1\to 2\left(4\right)$	$0\to 3^{\prime }/2\to 3\left(6\right)$	$0\to 4^{\prime }/3\to 4\left(8\right)$
2	0.2	−0.1031	-/-	-/-	-/-
3	0.2	−0.1031	−0.1012/−0.1053	-/-	-/-
4	0.2	−0.1031	−0.1012/−0.1053	−0.1006/−0.1061	-/-
5	0.2	−0.1031	−0.1012/−0.1053	−0.1006/−0.1061	−0.1003/−0.1065
2	3.0	−0.8837	-/-	-/-	-/-
3	3.0	−0.8837	−0.8832/−0.9414	-/-	-/-
4	3.0	−0.8837	−0.8832/−0.9414	−0.8828/−0.9607	-/-
5	3.0	−0.8837	−0.8832/−0.9414	−0.8828/−0.9607	−0.8824/−0.9703

**Table 3:** Change in free energy per added bond ($\Delta A_{N}\left(i\to j\right)/\Delta N_{b}\left(i\to j\right)$) between states i,j according to Figure 8 for the construction schemes (A, B) in case of a lattice of size N=3. Two different temperatures β={0.2,3.0} are considered. Each column contains two free energy values of the corresponding steps from both schemes where the same number of bonds are added (separated by “/”) to allow for a direct comparison. The value in brackets refers to the number of added bonds per step ($\Delta N_{b}$).

β	0→1B/1A→2A(2)	0→1A/1B→2B(4)
0.2	−0.1031/−0.1113	−0.1012/−0.1053
3.0	−0.8837/−1.0000	−0.8832/−0.9414

## Data Availability

The free energy source code used is publicly accessible at https://github.com/Thrawn1985/2D-Partition-Function (accessed on 21 April 2023). Input files for the code as well as Python-based analysis scripts used in this study are available upon reasonable request from the main author.

## Abbreviations

The following abbreviations are used in this manuscript:

CSV	Comma-separated values
DOS	Density of state
FBC	Free boundary conditions
PBC	Periodic boundary conditions
RMSE	Root mean square error

## Appendix A

### Appendix A.1

Figure A1 shows the residual free energy difference as function of bond fraction for various lattice sizes at constant temperature (β=1.133) for both schemes. In both cases, as *N* increases, the isothermes obviously decline and the set converges asymptotically to an (individual) limiting curve. The curves for scheme B are significantly smoother in the range of small $f_{b}$, since, as already mentioned, the resolution is much higher here compared to scheme A, i.e., A produces larger changes in $f_{b}$ in this range.

In the limit N→∞, the finite difference quotient defined in Equation (10) becomes a differential quotient $\left(da^{\mathrm{res}}/df_{b}\right)$. When plotted as function of $f_{b}$ as done in Figure A1, the area under the curve corresponds to the residual free energy difference between initial and end state of the construction schemes:

(A1a) $$\begin{array}{cc} I_{\infty } & =\int_{0}^{1}\left(\frac{da^{\mathrm{res}}}{df_{b}}\right)df_{b}=\Delta a_{\infty ,0\to 1}^{\mathrm{res}}=a_{\infty ,1}^{\mathrm{res}}-a_{\infty ,0}^{\mathrm{res}} \end{array}$$

with

(A1b) $$\begin{array}{cc} a_{\infty ,0}^{\mathrm{res}} & =a_{\infty }^{\mathrm{res}}\left(f_{b}=0\right)\overset{\mathrm{Equation}\;(5)}{\underset{N\to \infty }{=}}-\frac{\ln\left(\cosh\beta \right)}{\beta } \end{array}$$

(A1c) $$\begin{array}{cc} a_{\infty ,1}^{\mathrm{res}} & =a_{\infty }^{\mathrm{res}}\left(f_{b}=1\right)=a_{\infty }+\frac{\ln2}{\beta }\;\;\;\;\;\;\;\;\;\;\;\;\;\;\;\;\;\;\;\; \end{array}$$

where the free energy per spin in the case of an infinitely large lattice ($a_{\infty }$ in Equation (A1c)) is given by Equation (9). These relations can be used to quantitatively assess the degree of convergence for the two schemes by numerically integrating the differential residual free energy curves (the integral is denoted as $I_{N,\mathrm{num}}$) and comparing the result with the theoretical value $I_{\infty }$ according to Equation (A1a). Results for scheme A and B are summarized in Table A1 and Table A2, respectively.

As can be seen, the set of curves for scheme B can be accurately modeled using the following approach based on generalized negative power functions (see black dashed curves in Figure A1B):

(A2) $$\frac{\Delta a^{\mathrm{res}}}{\Delta f_{b}}\approx h_{N,B}\left(f_{b}\right)=\frac{\hat{a}}{{\left(f_{b}\right)}^{\hat{b}}}+\hat{c}$$

with three adjustable model parameters ($\hat{a}$, $\hat{b}$, $\hat{c}$). Optimized parameters as well as best fit results are given in Table A2. The model parameters were obtained by nonlinear least-squares fitting using the curve_fit package from the SciPy (version 1.11.2) optimize module, with parameter uncertainties estimated as the square roots of the diagonal elements of the covariance matrix. It should be noted that such a simple fit approach is not applicable for scheme A in contrast. The model approach in Equation (A2) offers an alternative and robust route to evaluate the degree of convergence in case of scheme B through analytical calculation of the relevant integral:

(A3) $$I_{N,\mathrm{ana}}=\int_{0}^{1}h_{N,B}\left(f_{b}\right)df_{b}=\frac{\hat{a}}{1-\hat{b}}+\hat{c}=\Delta {\hat{a}}_{\infty ,0\to 1}^{\mathrm{res}}$$

$\Delta {\hat{a}}_{\infty ,0\to 1}^{\mathrm{res}}$ is an approximation (due to the fitting step) for the true residual free energy difference $\Delta a_{\infty ,0\to 1}^{\mathrm{res}}$ defined in Equation (A1a). As revealed by the relative percentage error ($\epsilon_{I}$) between the theoretical and estimated areas of the residual free energy curves (last column in Table A1 and Table A2), scheme B exhibits superior convergence behavior compared to A. For B, the relative deviation is already on the order of 3% for a lattice with N=50, whereas for A this is not achieved until N=100. Construction scheme B also has the advantage that it can be approximated very accurately using the model Equation (A2).

The observed behavior is also valid for other temperatures, as can be seen in Figure A2. In contrast to the previous case, the various residual free energy curves were not evaluated at a single β but at (slightly) different values, in the form of the individual heat capacity peak temperature $\beta_{\max}\left(N\right)$ for each lattice size *N* according to Equation (2b). This means, as the lattice size increases, the temperature approaches the critical temperature $\beta_{c}\approx 0.441$ of the infinitely large lattice. Again, curves associated with scheme B can be well approximated by Equation (A2). As can be seen, the curves for N=50 and N=100 in the case of B are highly similar, which suggests rapid convergence. This superior convergence of scheme B compared to A is also confirmed by the values of the relative percentage error $\epsilon_{I}$ in Table A1 and Table A2. To ensure a meaningful comparison, the theoretical reference value $I_{\infty }$ according to Equation (A1a) was evaluated at $\beta_{c}$.

**Table A1:** Convergence behavior for construction scheme A for the free energy data presented in Figure A1A and Figure A2A, evaluated for lattice sizes 5≤N≤100. Data in the upper row refer to β=1.133; data in the lower row refer to heat capacity peak temperatures $\beta_{\max}\left(N\right)$ according to Equation (2b). $I_{N,\mathrm{num}}$ was estimated from numerical integration (trapezoidal rule) of the discrete data points. Convergence behavior is assessed by the relative percentage error $\epsilon_{I}\left(\%\right)=|I_{N,\mathrm{num}}-I_{\infty }\left|/\right|I_{\infty }|\cdot 100\%$ where $I_{\infty }$ was calculated from Equation (A1a–c) at β=1.133 ($I_{\infty }=-0.9131$) and $\beta_{c}=\ln\left(1+\sqrt{2}\right)/2\approx 0.441$ ($I_{\infty }=-0.3232$).

N	β	$I_{N,\mathrm{num}}$	$\epsilon_{I}\left(\%\right)$
5	1.133	−0.4073	55.390
20	1.133	−0.7717	15.486
50	1.133	−0.8555	6.309
100	1.133	−0.8841	3.172
5	0.5675	−0.2082	35.579
20	0.4703	−0.3040	5.933
50	0.4521	−0.3164	2.097
100	0.4461	−0.3197	1.089

### Appendix A.2

**Table A2:** Convergence behavior for construction scheme B for the free energy data presented in Figure A1B and Figure A2B, evaluated for lattice sizes 5≤N≤100. Data in the upper row refer to β=1.133; data in the lower row refer to heat capacity peak temperatures $\beta_{\max}\left(N\right)$ according to Equation (2b). Dependence of the optimized model parameters $\hat{a},\hat{b},\hat{c}$ of function $h_{N,B}\left(f_{b}\right)$ according to Equation (A2) on the lattice size *N* evaluated at various β. Metrics for assessing the quality of the fit: Coefficient of determination ($R^{2}$) and root mean square error (RMSE). $I_{N,\mathrm{num}}$ was estimated from numerical integration (trapezoidal rule) of the discrete data points. $I_{N,\mathrm{ana}}$ was calculated from Equation (A3). Convergence behavior is assessed by the relative percentage error $\epsilon_{I}\left(\%\right)=|I_{N,\mathrm{ana}}-I_{\infty }\left|/\right|I_{\infty }|\cdot 100\%$ where $I_{\infty }$ was calculated from Equation (A1a–c) at β=1.133 ($I_{\infty }=-0.9131$) and $\beta_{c}=\ln\left(1+\sqrt{2}\right)/2\approx 0.441$ ($I_{\infty }=-0.3232$).

N	β	$\hat{a}$	$\hat{b}$	$\hat{c}$	$R^{2}$	RMSE	$I_{N,\mathrm{num}}$	$I_{N,\mathrm{ana}}$	$\epsilon_{I}\left(\%\right)$
5	1.133	0.0733±0.0065	0.5072±0.0261	−0.7433±0.0071	0.9999	$4.67\;\times \;10^{-4}$	−0.5639	−0.5946	34.874
20	1.133	0.0135±0.0003	0.5656±0.0042	−0.8659±0.0005	0.9999	$6.64\;\times \;10^{-4}$	−0.8330	−0.8347	8.580
50	1.133	0.0049±0.0001	0.5642±0.0017	−0.8935±0.0001	0.9999	$4.40\;\times \;10^{-4}$	−0.8819	−0.8822	3.386
100	1.133	0.0023±0.0000	0.5604±0.0011	−0.9030±0.0001	0.9999	$3.60\;\times \;10^{-4}$	−0.8977	−0.8977	1.687
5	0.5675	0.2344±0.0222	0.1346±0.0109	−0.5827±0.0223	1.0000	$1.14\;\times \;10^{-4}$	−0.2889	−0.3119	3.508
20	0.4703	0.0207±0.0005	0.3173±0.0035	−0.3613±0.0005	0.9999	$2.43\;\times \;10^{-4}$	−0.3298	−0.3310	2.402
50	0.4521	0.0058±0.0001	0.3824±0.0026	−0.3367±0.0002	0.9997	$2.75\;\times \;10^{-4}$	−0.3270	−0.3272	1.238
100	0.4461	0.0025±0.0000	0.4109±0.0020	−0.3294±0.0001	0.9994	$2.64\;\times \;10^{-4}$	−0.3250	−0.3251	0.585
